# Photon activated therapy (PAT) using monochromatic Synchrotron x-rays and iron oxide nanoparticles in a mouse tumor model: feasibility study of PAT for the treatment of superficial malignancy

**DOI:** 10.1186/1748-717X-7-184

**Published:** 2012-10-31

**Authors:** Ki-Hong Kim, Hong-Tae Kim, Sung-Hwan Park, Jae-Hong Lim, Jong-Ki Kim

**Affiliations:** 1 Department of Neurosurgery, School of Medicine, Catholic University of Daegu, 3056-6 Taemyung 4 Dong, Nam-Ku, Daegu City, 706-034, Korea; 2 Department of Biomedical Engineering, Catholic University of Daegu, 3056-6 Taemyung 4 Dong, Nam-Ku, Daegu City 706-034, Korea; 3 Department of Optometry and Visual Science, College of Medical Science, Catholic University of Daegu, 13-13 Hayang-Ro, Hayang-Eup, Gyeongsan, 712-702, Korea; 4 Department of Anatomy, Catholic University of Daegu, 3056-6 Taemyung 4 Dong, Nam-Ku, Daegu City, 706-034, Korea; 5 Departments of Neurosurgery, School of Medicine, Catholic University of Daegu, 3056-6 Taemyung 4 Dong, Nam-Ku, Daegu City, 706-034, Korea; 6 Pohang Accelerator Laboratory, POSTECH, Hyojadong San 31, Pohang, 790-784, Korea

**Keywords:** Photon activated therapy, Iron oxide nanoparticles, Auger electron, Synchrotron monochromatic x-rays, Superficial malignancy

## Abstract

**Background:**

X-rays are known to interact with metallic nanoparticles, producing photoelectric species as radiosensitizing effects, and have been exploited *in vivo* mainly with gold nanoparticles. The purpose of this study was to investigate the potential of sensitizing effect of iron oxide nanoparticles for photon activated therapy.

**Methods:**

X-rays photon activated therapy (PAT) was studied by treating CT26 tumor cells and CT26 tumor-bearing mice loaded with 13-nm diameter FeO NP, and irradiating them at 7.1 keV near the Fe K-edge using synchrotron x-rays radiation. Survival of cells was determined by MTT assay, and tumor regression assay was performed for in vivo model experiment. The results of PAT treated groups were compared with x-rays alone control groups.

**Results:**

A more significant reduction in viability and damage was observed in the FeO NP-treated irradiated cells, compared to the radiation alone group (*p* < 0.04). Injection of FeO NP (100 mg/kg) 30 min prior to irradiation elevated the tumor concentration of magnetite to 40 μg of Fe/g tissue, with a tumor-to-muscle ratio of 17.4. The group receiving FeO NP and radiation of 10 Gy showed 80% complete tumor regression (CTR) after 15–35 days and relapse-free survival for up to 6 months, compared to the control group, which showed growth retardation, resulting in 80% fatality. The group receiving radiation of 40 Gy showed 100% CTR in all cases irrespective of the presence of FeO NP, but CTR was achieved earlier in the PAT-treated group compared with the radiation alone group.

**Conclusions:**

An iron oxide nanoparticle enhanced therapeutic effect with relatively low tissue concentration of iron and 10 Gy of monochromatic X-rays. Since 7.1 keV X-rays is attenuated very sharply in the tissue, FeO NP-PAT may have promise as a potent treatment option for superficial malignancies in the skin, like chest wall recurrence of breast cancer.

## Background

X-rays are known to interact with high-Z elements, and radiosensitizing effects have been explored for biomedical applications [1,2]. X-rays photoabsorption in tumours containing high Z elements can be effectively enhanced by tuning the energy of X-rays to inner-shell absorption edge of the Z-element, followed by Auger de-excitation processes, inducing instantaneous emission of Auger electrons. Low-energy Auger electrons can directly cause breaks in single- and double-stranded DNA and damage other cellular components by indirect induction of reactive oxygen species, which are responsible for dose enhancement to the tissue [3-6]. The therapeutic application of these phenomena has been exploited *in vivo* mainly with gold nanoparticles [7,8] or metal chelate compounds [9-15]. Although this concept has been widely accepted as radiosensitization with a polychromatic x-rays source, recent work has investigated the use of resonant x-rays therapy (RXT) [3] or photon activation therapy [16] as an alternative when exploiting monochromatic x-rays sources. For potent medical applications, high-energy monochromatic x-rays have considerable advantages in reducing the non-target dose by eliminating unnecessary lower energy x-rays absorption by the tissue, suggesting their ability to reach various target organs depending on the penetration depth of a particular x-ray energy level. In this regard, gold [4-7], platinum [9,10], gadolinium [15], and iodine [11] have been investigated for the therapeutic application of their relatively high K-edge x-rays energies (80, 78, 50, and 33 keV, respectively) in various cancer models to deeply embedded tumors for actual clinical environment.

Radio sensitizing effect of super paramagnetic nanoparticles was examined extensively with respect to broad band x-rays in previous report [17]. High energy X-ray from LINAC source did not show dose enhancement significantly upon irradiation on iron nanoparticles. It was known that K-shell resonance complexes enhanced total x-rays absorption by larger factors up to ~10^3^ for Au, and smaller but significant factors ~10 for Fe, near corresponding K-edge [3]. The energy of the Fe K_α_-X-rays is relatively small compared to that of high-Z elements, and therefore, it penetrates only a few millimeters into the tissue. Because of variations in the anatomical position, size, and depth of the tumor, each x-rays energy band has specific applications or limitations in a given topographical area. Administration of magnetite nanoparticles into the tumor combined with irradiation of monochromatic x-rays may be suitable for the treatment of superficial or disseminated malignant diseases including cutaneous neoplasm or lymphoma, peritoneal metastasis and chest wall recurrence of breast cancer.

In this PAT study with Fe-Kα photon, we found a remarkable increase in complete-tumor-regression (CTR) with PAT dose enhancement effect compared with x-rays irradiation alone, and combinatorial therapeutic effective dose of monochromatic x-rays and tumoral uptake concentration of iron nanoparticle prior to photon activation. Importantly, PAT effect led to CTR with relatively low tissue concentration of iron nanoparticles and low x-rays dose compared with previously reported radio sensitization effect with gold nanoparticles. The result of this study suggests a broadly applicable way to manipulate the tissue concentration of nanoparticle and radiation dose depending on tumor growth patterns for enhanced radiotherapy performance.

## Methods

### Metal nanoparticles

Alginate-coated super paramagnetic magnetite nanoparticles (FeO NP) were synthesized by insonating ferrous and ferric salt solutions, as reported previously [18,19]. Briefly, FeCl_2_·4H_2_O (1.72 g) and FeCl_3_·6H_2_O (4.70 g) (8.65 mmol Fe^2+^/17.30 mmol Fe^3+^) were dissolved in 80 ml of distilled water. A black magnetic oxide precipitate was obtained by heating the solution to 80°C in argon atmosphere, increasing the pH to 10 by adding 28~30% ammonium hydroxide to the water, and insonating the mixed iron solution with 20-kHz ultrasound at a power output of 140 W for 1 h. Alginate was used to coat the nanoparticle surface to disperse the particles. Briefly, 2 g of magnetite nanoparticles were dispersed in 60 ml of saline and 25 ml of alginic acid solution by heating the solution to 80°C while insonating at power output of 50 W for 30 min under nitrogen gas with continuous stirring. The particles were purified by washing with saline while being exposed to a strong neodymium magnet (magnetic field density; *B*_*r*_ = 11,000 Gauss). Finally, a ferrofluid containing 25 mg/ml FeO NP was obtained.

### Transmission electron microscopy (TEM) studies

The average particle size, size distribution, and morphology of FeO-NP were examined using a Zeiss 902 transmission electron microscope (Carl Zeiss Pte., Ltd., Oberkochen, Germany) at a voltage of 80 kV. The aqueous dispersion of the particles was drop casted onto a carbon-coated copper grid, and the grid was air dried at room temperature before microscopic observation.

### Cellular uptake and distribution of FeO NP

Cellular uptake of FeO NP was measured as a function of the incubating concentration by using an inductively coupled plasma mass spectrometer (ICP-MS, Thermo Jarrell Ash ARIS-AP; USA). A total of 5 × 10^6^ CT26 colon adenocarcinoma cells were plated in each Petri dish containing different concentrations of FeO-NP solution. After overnight incubation, each dish was washed out with phosphate buffer to remove nanoparticles that were not taken up in cell. Data are presented as the average of the uptake density of 10^6^ cells per incubating dose after harvesting cells for ICP-MS measurements. Cellular distribution of FeO NP was assayed using phase contrast microscopy (Carl Zeiss, Germany). CT26 colon adenocarcinoma or C6 glioma cells were plated at a density of 1 × 10^5^ cells/well in Kapton film and incubated with 1 mg/ml nanoparticle solution.

### In vitro cytotoxicity studies of FeO NP

To determine cell cytotoxicity/viability, CT26 tumor cells were plated at a density of 1 × 10^4^ cells/well in 96-well plates at 37°C in 5% CO_2_. After 24 h of culture, the medium in the wells was replaced with fresh medium containing nanoparticles in a concentration range of 0–2.0 mg/ml. After 24 h of incubation with nanoparticles, each well was refreshed with new medium after washing out. 20 μl of MTT dye solution (5 mg/ml in phosphate buffer, pH-7.4) was added to each well. After 4 h of incubation at 37°C, the medium was removed, and the formazan crystals were solubilized with 200 μl of dimethylsulphoxide (DMSO) with vigorous mixing to dissolve the reacted dye. After 15 min, the absorbance of each well was read on a multi scanner auto reader (Flurostar optima; BMG Labtech, Germany) at 570 nm. The spectrophotometer was calibrated to zero absorbance by using culture medium without cells.

### Animal models

A CT26 tumor model was prepared by using Balb/C mice. A 0.05-ml cell suspension containing 1 × 10^5^ CT26 colon adenocarcinoma cells (ATCC) was subcutaneously injected into the thigh. After a 7–10-day interval to allow tumor growth, a typical diameter at the time of treatment was measured as much as 6–11 mm.

### Tumoral uptake of FeO NP

To measure nanoparticle uptake in the tumors, nanoparticle doses of 100 or 300 mg/kg body weight were administered to the CT26 tumor models via the tail vein 30 min prior to surgical removal of the tumors. In addition normal muscle was also sampled to measure tumor-to-muscle ratio. Tumor and normal muscle samples were placed in tared vials and analyzed for iron using ICP-MS spectrometry.

### Radiation experiments

**Figure 1:**
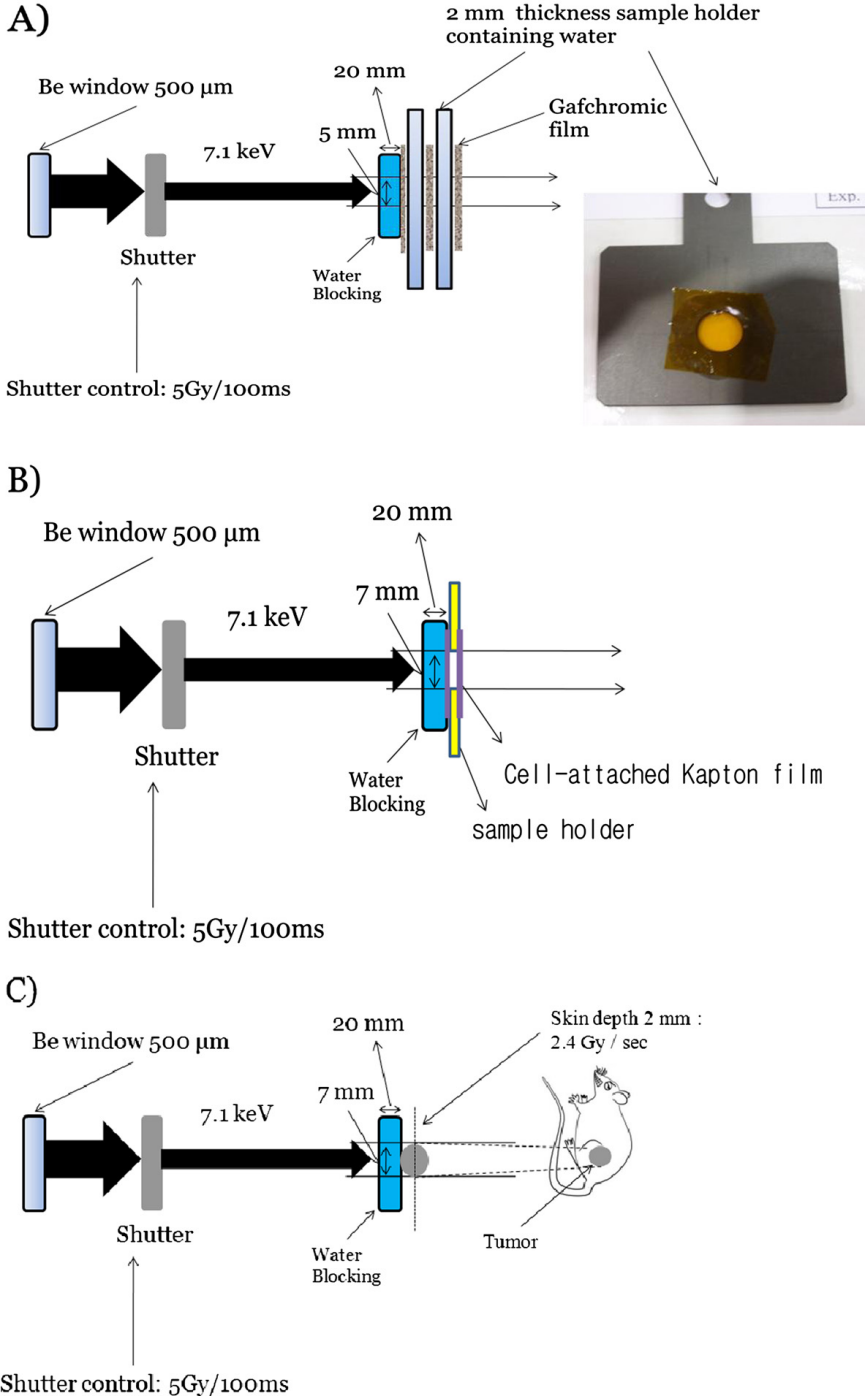
**Schematic diagrams of radiation dose calibration (A), *in vitro *(B) and *in vivo *(C) PAT experiments.** A sample holder containing the cells was placed vertically and facing the horizontal beam. Anesthetized mice were placed on the sample stage. Radiation dose was delivered with either 5.0 Gy/100 ms at the surface of sample holder for cell or 2.4 Gy/s at a skin depth of 2 mm for animal model using a fast shutter with time resolution of 10 ms. For entering dose of 50 Gy/s, 0.115 Gy/s could be estimated as an exit dose rate in 4 mm-depth tumor, and 2.4 Gy/s in 2 mm-depth cell sample holder, respectively. An acryl block with a hole was used to prevent unnecessary exposure of either Gafchromic film or the normal tissue surrounding the tumor.

The cells and animal models were irradiated with 7.1 KeV monoenergetic synchrotron X-rays at the 1B2 beamline of Pohang Accelerator Laboratory (Pohang, Korea) to activate inner-shell ionization of FeO NP by maximizing absorption efficiency near the Fe K-edge value, thereby effectively generating Auger cascades. X-rays energy was generated by a multilayer monochromator (Mo/B/4C) which was calibrated with the Fe-Kα line of iron reference materials. Band width of monochromatic X-rays was observed as 140 eV at 7.1 keV. Measurement of dose versus depth was performed using a radio chromic film HD-810 and 2 mm-thickness water phantom (sample holder containing water) as shown in Figure 1A. Each 2.0 cm × 2.0 cm film sample was sandwiched between adjacent slab faces. Radiation dose was measured at selected depths; 50 Gy/s as entering dose, 2.4 Gy/s at 2mm, 0.115 Gy/s at 4 mm, 0.0012 Gy/s at 6 mm. Radiation dose was delivered with either 5.0 Gy/100 ms at the surface of sample holder for cell or 2.4 Gy/s at a skin depth of 2 mm for animal model using a fast shutter with time resolution of 10 ms. For entering dose of 50 Gy/s, 0.115 Gy/s could be estimated as an exit dose rate in 4 mm-depth tumor, and 2.4 Gy/s in 2 mm-depth cell sample holder, respectively. Therefore, there will be large gradient in tumor dose due to high tissue attenuation of 7.1 keV x-rays even in 4 mm-depth tumor upon delivering 5 Gy to the half of tumor depth. Animal model that had a tumor depth more than 5 mm was excluded in this study.

The schematic drawing of the x-rays irradiation on the *in vitro* samples is shown in Figure 1B. Anchored cells in a Kapton film were irradiated in a specifically designed well device filled with media, which was placed vertically facing the horizontal beam. Two different groups of cells were prepared, namely, an experimental group treated with FeO NP and a control group that was not treated with FeO NP. Each group was irradiated with 2 different x-rays doses, 5 and 20 Gy as entering dose at the surface of sample holder, thereby generating 4 working groups. Measurements were made in 3 replicates for each group.

Synchrotron x-rays beam irradiation in animal models was investigated according to the experimental setup presented in Figure 1C. Four working groups of CT26 models were generated from 2 mice groups given FeO NP with a dose of 100 mg/kg body weight or 2 mice groups without nanoparticles. Mice were anesthetized by intra peritoneal injection of ketamine (20 mg/kg) and xylazine (18.4 mg/kg). Fifty micro liters of FeO NP saline solution were intravenously injected 30 min prior to irradiation. Two trial radiation doses, low (LD) and high dose (HD), were chosen based on a dose finding procedure and rationale of conventional therapeutic dose of solid tumor, respectively. LD was chosen such that it was not intense enough to produce tumor regression under X-rays alone, and thus potent PAT effect may be emerged as enhanced tumor dose. Trial 5-Gy irradiation alone and further repeated treatment 10 day after first trial did not show any tumor regression. Thus 10 Gy was set to LD, whereas 40 Gy, as a typical therapeutic dose for solid tumor, was given as HD. Mice were irradiated with 7.1-keV x-rays using a monochromator to deliver Fe K-edge resonance energy of total 10 Gy or 40 Gy at a dose rate of 2.4 Gy/s to the sample, as shown in Figure 1C. Thus treatment was repeated with a trial dose (LD or HD), effectively dividing total irradiation dose into 2 fractions by delivering either 5 or 20 Gy per treatment separated by 10 days. All mice after first PAT treatment received same dose of FeO NP as given first prior to second PAT. An acryl block with a hole was used to prevent unnecessary exposure of the normal tissue surrounding the tumor.

### Cytotoxicity assay of monochromatic x-rays-irradiated cells

The cell viability was measured in the experimental groups by using the MTT assay. At 24 h after irradiation, a working concentration of MTT solution (10 μl in 100 μl of DMEM media) was added to each well. After continued incubation for 4 h, the supernatant was removed, and 100 μg of DMSO was added. When the purple crystals were completely dissolved, the absorbance of the converted dye was measured at a wavelength of 570 nm with a 96-well multiscanner autoreader (Flurostar optima; BMG Labtech, Germany).

### Cytologic examination of irradiated cells

C6-glioma cells were plated at a density of 1 × 10^5^ cells/well in Kapton film, and then prepared in a specially designed aluminum well plate for microscopic examination, using a method similar to the one described for the MTT assay. Irradiated cells were stabilized overnight prior to a change of the culture medium, and stained with acridine orange (AO) or ethidium bromide (EB) to measure viability with a fluorescent microscope (Olympus, Japan), and to observe cell morphology with phase contrast microscopy (Carl Zeiss, Germany).

### Tumor regression assay and statistical analysis

The dimensions of the tumor were measured with vernier calipers. The tumor shape was assumed to approximate a spheroid. The volume was calculated according to the standard formula for volume of an ellipsoid [20],

4π/3×x/2×y/2×z/2

where x, z are inter orthogonal lengths, and y is typically tumor depth.

Tumor volumes were measured daily after treatment either until CTR was observed for PAT-treated mice or until death for untreated mice otherwise 35 th days for X-rays alone control mice. Comparison of tumor growth was performed using repeated-measure ANOVA. The impact of irradiation and FeO NP on tumor growth was analyzed by calculating a mean tumor volume growth rate (TVGR) as a general linear model, and the differences among groups were compared by the Games-Howell method. The significance level was set at *P* < 0.05. A TVGR of each group was calculated during a period of 10–30 days post the treatment in which growth behaviors appeared distinctive among groups.

TVGR = ([tumor volume at 30 day − tumor volume at 10 day]/20 days) × 100 (%).

Survival of CTR mice in any group were monitored during 6 month follow-up.

## Results

### Size distribution studies by TEM measurements

The average size of the particles was determined by TEM, by using measurements of the size of approximately 200 particles. The particles had a globular shape and an approximate size of 10.6 nm with a standard deviation of 0.78 nm. The size of the particles after coating was 13–15 nm in diameter.

### Cellular uptake and dark cytotoxicity of magnetic nanoparticles

**Figure 2:**
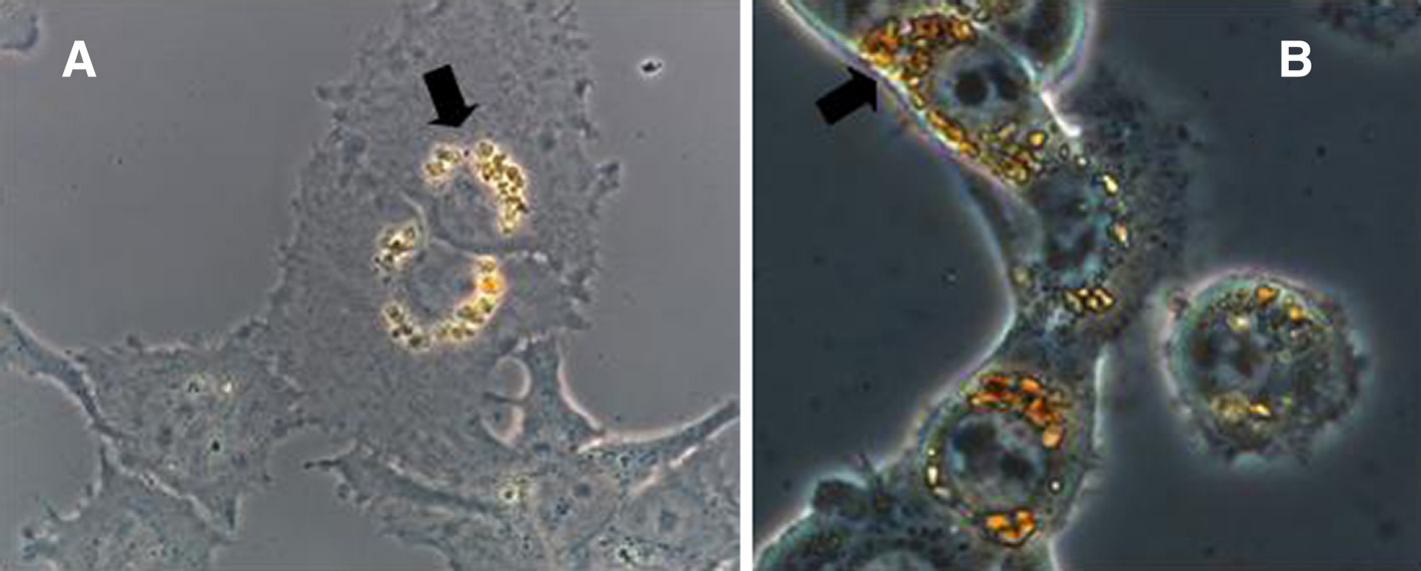
**Optical microscopy study of the cellular distribution of FeO NP in C6 glioma cells (A) and CT 26 colon adenocarcinoma cells (B).** Magnetite nanoparticles were distributed in the perinuclear membrane in both the cell lines.

**Figure 3:**
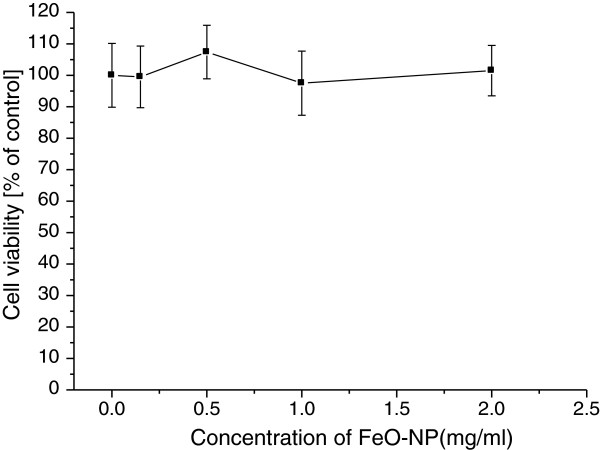
**Dark cytotoxicity results of magnetite nanoparticles by MTT assay.** Cellular uptake of FeO NP showed no cytotoxic effects, and the cells remained more than 97% viable relative to the control at FeO NP concentrations as high as 2.0 mg/ml.

FeO NP was distributed in the perinuclear membrane of both CT26 colon adenocarcinoma and C6 glioma cells as shown in Figure 2A-B. Cellular uptake of FeO NP increased with incubating dose in the range of 0.2 - 2 mg/ml, as summarized in Table 1. Data are presented as μg of FeO NP/1 × 10^6^ cells, typically 140 μg per 10^6^ cells when incubated with 1 mg/ml nanoparticle solution. FeO NP showed no cytotoxic effects, and cells remained more than 97% viable relative to the control at concentrations as high as 2.0 mg/ml, as shown in Figure 3.

**Table 1:** Results of cellular uptake following overnight incubation of metal nanoparticles

		**Incubating Dose**	
Group	0.20 mg/ml	1.00 mg/ml	2.00 mg/ml
FeO NP (μg of Fe/10^6^ cells)	30.7 ± 6.65	139.7 ± 16.30	168.8 ± 12.56

5 × 10^6^ CT-26 cells were plated in each Petri dish containing different concentrations of MNP solution. Measured data are presented as the average uptake density per 10^6^ cells in each incubating dose after harvesting cells for ICP measurements.

### Cytotoxic effects of resonant x-rays irradiation

**Figure 4:**
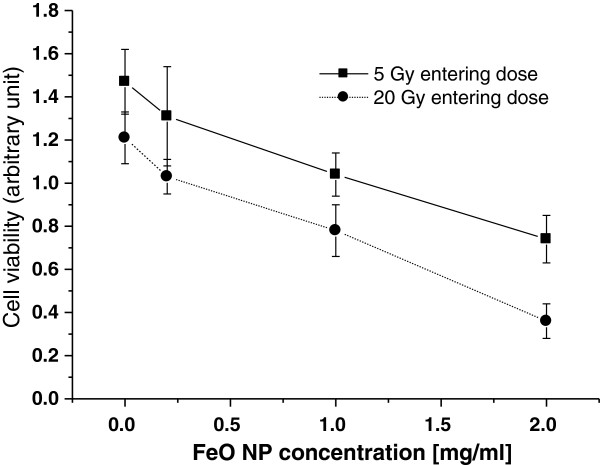
**The viabilities of irradiated cells as function of FeO NP concentration.** The viabilities of PAT-treated cells were displayed as arbitrary unit representing absorbance at 570 nm. The viability of irradiated cells was reduced in a nanoparticle dose-dependent manner at both x-ray doses. The cell viability loss increased by either 50% or 70% in the cells treated with 2.0 mg FeO NP/ml at both x-ray doses.

In the cell-attached Kapton film, actual 100 ms exposure delivered 0.24 Gy upon irradiation with 5 Gy/100 ms, or 0.96 Gy under exposure of 400 ms. The survival of irradiated CT26 cells treated with nanoparticles was measured and compared with radiation alone control cells. The viability of irradiated cells was reduced in a nanoparticle dose-dependent manner at both x-ray doses, as shown in Figure 4.

### Cytological studies

**Figure 5:**
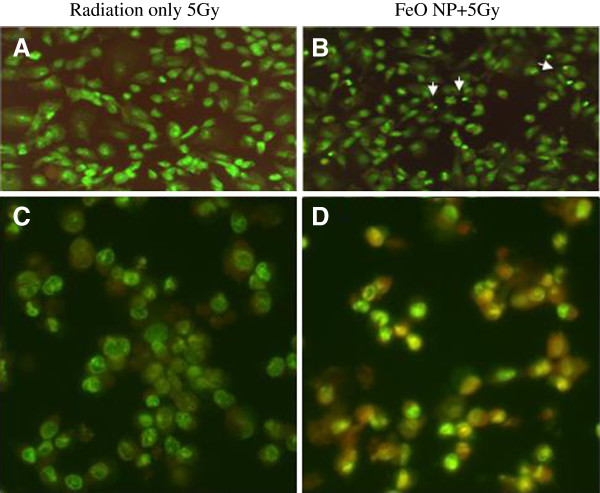
**Fluorescence microscopic image of radiation only (RO-5Gy) control group (A, C) and FeO-NP+5Gy radiation group (B, D); C and D are expanded view of A or B, respectively to show the cellular change in fluorescent color more clearly.** The damaged shrunken nuclei indicate apoptosis, which was more frequently observed in (**B**) compared to (**A**). AO stained the damaged DNA, which altered the fluorescence. EB did not penetrate or showed less penetration into the cell nucleus in the control group. In contrast, EB entered the nuclei of the cells in the FeO-NP+5Gy group (**D**), reflecting the presence of more nuclear damage than the RO-5Gy group (**C**).

To determine the effect of x-rays irradiation on the cells treated with magnetic nanoparticles, the cells were co-stained with EB or AO. AO staining identified damaged DNA in the experimental group by showing changes in fluorescence. EB did not show positive nuclear staining in the control group, while it showed significant staining of the nucleus in cells treated with FeO NP and 10 Gy radiation, indicating the extent of DNA damage in the cells of the experimental group compared to the control groups. This was observed as a color change, resulting in a reddish stain as seen in Figure 5. Overall, the cell nuclei appeared shrunken, reflecting nuclear damage and apoptosis (Figure 5). Detection of nuclear damage by EB and AO staining was more common in the experimental group than in the control group, which was consistent with the results of the MTT assay and the perinuclear distribution of FeO NP.

### Tumor uptake and in vivo therapeutic effect of PAT

ICP-MS data are summarized in Table 2. Tumor concentrations of FeO NP 30 min after injection with a dose of 100 mg/kg were 40.3 ± 8.2 μg Fe/g tissue, while the corresponding muscle concentrations were 6.5 μg Fe/g tissue. The tumor-to-muscle FeO NP ratio was 17.4 after 30 min post-injection, enabling tumor-selective activation of FeNP by the monochromatic X-rays and enhanced tumor dose deposition. Quantitative data demonstrated that tumor uptake increased with the injection dose of FeO NP. Similar tissue-to-injection FeO NP ratio was obtained from the administration with two injection doses, 100 mg/kg body weight and 300 mg/kg body weight. When iron nanoparticles were injected, less than 1% of the injected dose was taken by tumor in a given time interval after injection.

**Table 2:** Results of tumor uptake 30 min following the injection of metal nanoparticles

			**Injection Dose**	
		Tissue	100 mg/kg	300 mg/kg
tissue	concentration(μg of Fe/g)	Tumor	40.30 ± 8.24	58.85 ± 12.30
		Muscle	2.30 ± 0.81	6.50 ± 3.80
	up take/injection (%)		0.55	0.40

Data are presented as average ± standard deviation (n = 3).

**Figure 6:**
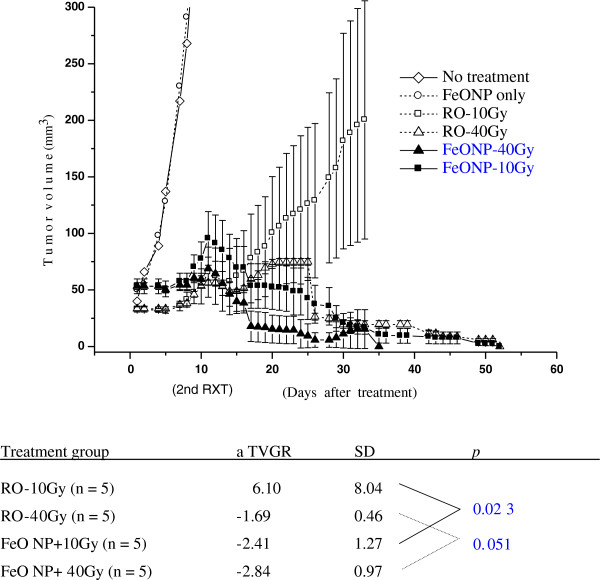
**Average tumor volume graphs of the following groups: no treatment (NOT; n = 3), FeO NP only (FeO NP only; n = 3), radiation only with 10 Gy (RO-10 Gy; n = 5), radiation only with 40 Gy (RO-40 Gy; n = 5), FeO NP injection (100 mg Fe/kg) followed by 10 Gy-irradiation (FeO NP-10Gy; n = 5), and FeO NP injection (100 mg Fe/kg) followed by 40-Gy irradiation (FeO NP-40 Gy; n = 5).** Comparison between the experimental groups or control group was done on the basis of the tumor growth curves. The average TVGR_10–30 days_ of the FeO NP-PAT group significantly differed from those of the control group (*p* < 0.04).

Administration of 100 mg of FeO NP/kg without radiation did not retard tumor growth and had no therapeutic effect. The therapeutic effects of PAT with FeO NP are shown in Figure 6. The low-dose radiation only group (RO-10Gy) showed a partial response as a decrease in tumor growth in the first 15 days post irradiation, followed by re-growth. The radiation only group irradiated with higher doses (RO-40Gy) demonstrated partial control of tumor growth until 25 days post irradiation, followed by a complete tumor response 50 days after treatment. The average rate of tumor growth was significantly different in the mice receiving only x-rays radiation and those receiving FeO NP followed by x-rays irradiation, as shown in Figure 6 (*p* < 0.04). The FeO NP-10Gy mice demonstrated much larger tumor volume regression (90%) 30 days after PAT and percent CTR (80%) at average 35 days post-treatment compared to the RO-10Gy mice, showing 20% CTR and growth retardation. The FeO NP-40Gy mice also showed same percent of tumor volume regression, but 100% CTR at 17–50 days post-treatment, which was a shorter time period compared to the RO-40Gy mice.

The CTR data of the treatment groups is summarized in Table 3. Dose-dependent increase in survival was observed from PAT treatment during 6 month follow-up. All CTR-mice survived for the six month follow-up without relapse, otherwise mostly died within 50 days after treatment.

**Table 3:** CTR incidence and 6-month survival rate in animals receiving treatment

**Group**	**RO-10Gy**	**FeO NP-10Gy**	**RO-40Gy**	**FeO NP-40Gy**
CTR incidence	1/5	4/5	5/5	5/5
6-month survival rate (%)	20	80	100	100

## Discussion

The CTR mice by treatment with FeO NP and Fe-Kα photon indicates that this protocol is able to produce PAT effect, enabling therapeutic enhancement at relatively lower x-rays dose despite large gradient in tumor dose. Enhanced ROS generation and energy-dependent migration mobility of related ROS molecules may be attributable to such enhancement under the inhomogeneous distribution of tumor dose [4]. The larger tumor volume regression and shorter period to CTR in mice receiving high-dose PAT compared to the x-rays alone suggested that PAT-associated effects were partly attributable to tumor regression even under high dose irradiation. Tumor growth behaviors after second treatment demonstrated distinctive features between growth retardation and tumor regression among the mice groups, which was demonstrated clearly in terms of different sign of TVGR_10-30_. Three mice groups, treated by 10 Gy+FeONP, 40 Gy alone or 40 Gy+FeONP, presenting 80%-100% CTR with negative TVGR_10-30_, exhibited similar decaying trends until CTR in their median growth curves despite spread-out CTR periods in 35–52 days. This may present variable tumor response depending on baseline tumor size to therapeutic effective dose combined x-rays and dose enhancement effect of photon activated nanoparticles.

The PAT effects under a low dose irradiation suggests not only dose enhancement within the tumor but also less side effects to the surrounding normal compared to therapeutic high-dose irradiation alone, effectively tumor-specific. Therefore, low-dose PAT can be exploited potentially to treat the tumor spreading in normal as long as iron nanoparticles are preferentially taken in tumor cell. External irradiation with monochromatic high-energy x-rays is a promising new method in radiotherapy to generate PAT effects in deep-embedded tumors under the normal tissue tolerance. In contrast, Fe-Kα x-rays, attenuated completely within a few millimeters, may have advantage to treat selectively superficial malignant diseases particularly near skin.

Theoretically, tumor dose enhancement upon photon activation is achieved through the irradiation of high-Z elements at their K-edge absorption energy, leading to emission of Auger electrons and x-rays photons, which results in the release of a large amount of energy in their immediate vicinity [9,16,21,22]. Our previous radiosensitization experiment on a tumor cell given FeO NP with high energy LINAC X-rays showed much less dose enhancement effect (only 10%) compared to tuned monochromatic X-rays irradiation (50–70%) [17]. This large difference in therapeutic efficiency suggested that therapeutic enhancement with FeO NP and its K-edge irradiation were related to photoelectric enhancement on iron nanoparticles.

In particular, irradiation of iron oxide nanoparticles with monochromatic x-rays causes more effective production of x-rays and Auger electrons by multiple inner shell ionization, because a 13-nm FeO NP contains more than 6000 Fe atoms and a significantly higher resonance attenuation coefficient for K_α_ transitions [3]. Since the energy of emitted x-rays photon is around 7.0 keV, these x-rays becomes attenuated within a few millimeter of tissue (Linear attenuation coefficient μ =17.1 cm^-1^, HVL=0.04 cm at 7 keV for water or soft tissue)[23], thereby potentially contributing to tumor dose. Previously, *in vivo* studies were performed either with a broadband x-rays source with gold NP or with monochromatic x-rays with metal chelate compounds. Only one Monte Carlo simulation has been done for the interaction of 68.4-keV monochromatic x-rays with gold NP in a tumor phantom study, which demonstrated a large dose enhancement from the release of Auger electrons and photons [21]. To the best of our knowledge, the present study is the first *in vivo* PAT study using monochromatic x-rays and metallic nanoparticles. Given that a high-Z nanoparticle is an atomic–molecular cluster, Auger decays from a single atom will lead to a cascade of photon-electron emissions that would impinge upon nearby atoms. Thus, further photoionization via an autoionization state can be produced, causing multiple ionizations resulting from the Auger cascades with a given incident x-rays energy. However, maximum resonance absorption cross-section and upward K-shell excitation can be achieved via monochromatic Kα photon source of sufficient intensity like synchrotron based X-rays source which is capable of high photon flux in excess of 10^9^. The K-shell resonance complexes enhanced the total x-rays absorption by a factor of ~10 for Fe at a specific resonant energy *E(K*_*α*_*)* relative to the background [3]. Although this resonance factor is smaller than that obtained with Au (10^3^), it is still large enough to produce Auger cascades through inner shell ionization (ISI) effectively. Mostly, photoelectric effects contribute to the ionization of the iron atoms in nanoparticles, enabling energy deposition in the tumor tissue, while the contribution from Compton scattering to the ionization would be negligible at this photon energy level. Although the mass percentage of FeO NP was less than 0.01% of the total exposed tissue mass, in the presence of FeO NP, only this fraction would be responsible for absorbing incident x-rays to produce photon-activating effects. However, the concentration of iron in tumor, 40 μg Fe/g tissue, was sufficient to demonstrate contrasting therapeutic efficacy, the CTR in FeO NP-10Gy mice and the growth retardation in RO-10Gy mice.

Nanoparticle distribution along nuclear envelope may present facilitated and frequent nuclear damage including DNA with short-range Auger electrons, which was well correlated with more frequent observation of shrunken nuclei and penetration of ethidium bromide/acridine orange into disrupted nucleus (showing altered fluorescence in Figure 5D compared with Figure 5C). Since PAT effect take place predominantly via Auger electrons [5], this may represent characteristic pattern of cellular damage in the PAT-treated tumor cell. The biological effects of Auger electrons with respect to their therapeutic effect are often explained from the viewpoint of the linear energy transfer (LET). The photon-activating process generates high-LET Auger electrons [24] through the interaction of metal nanoparticles with low-LET x-rays radiation [1].

A number of nanoparticle-mediated thermal therapies on CT26 tumor model have been attempted to treat solid tumor using iron [25] or gold nanoparticles [26,27] by magnetic induction or laser-driven surface plasmon activation, respectively. One of major obstacles in magnetic hyperthermia was requirement of too large therapeutic effective drug dose in consideration of systemic injection despite successful demonstration of therapeutic efficacy. For instance 5 mg Fe/g tissue for magnetic hyperthermia cannot be easily achievable by intravenous injection of iron nanoparticles, instead direct intratumoral injection was performed in most application. Gold nanoparticles showed efficient photothermal coupling with large absorption cross sections, and their high thermal conductivity couples this heat to the surrounding tissue. These resulted in hyperthermic temperature with submiligram tissue gold concentration, and thus 90% complete tumor regression with 4 W/cm^2^ that may cause potent thermal damage on normal, but 42% CTR with lower fluence rate of laser power. Therefore main therapeutic target of photothermal therapy would be relatively small-sized solid tumor nearby skin with near infrared light (2–3 cm as penetration depth) as optical window for maximum tissue penetration.

Similar problem, potent normal skin damage, associated with high entrance dose in single dose treatment of Fe Kα-PAT can be minimized by multiple fractionations to distribute tolerable dose to normal skin but keeping enhanced tumor dose by PAT effect. In conclusion, magnetite nanoparticles combined with monochromatic K-edge x-rays show promise as a potent PAT method, particularly for the treatment of superficial malignancies.

## Misc

Gi-Hwan Choi and Seung-Jun Seo both authors equally contributed to this work

## Competing interests

The authors declare that they have no competing interests.

## Authors’ contributions

GC performed experiments. SS prepared animal model and measured dosimetry. Both GC and SS equally contributed to this work. KK helped to measure X-rays radiation and make sample holder. HK performed assay fluorescence microscopy and MTT assay. SP participated importantly in the conception of the study and helped to draft the manuscript. JL helped to prepare monochromatic X-rays and calibrate radiation dose. JK conceived of the study and drafted the manuscript. All authors read and approved the final manuscript.
